# Drop-On-Powder 3D Printing of Tablets with an Anti-Cancer Drug, 5-Fluorouracil

**DOI:** 10.3390/pharmaceutics11040150

**Published:** 2019-04-01

**Authors:** Kejing Shi, Deck K. Tan, Ali Nokhodchi, Mohammed Maniruzzaman

**Affiliations:** 1 School of Life Sciences, University of Sussex, Falmer, Brighton BN1 9QJ, UK; K.Shi@sussex.ac.uk (K.S.); D.Tan@sussex.ac.uk (D.K.T.); 2 Drug Applied Research Center and Faculty of Pharmacy, Tabriz University of Medical Sciences, 5166/15731 Tabriz, Iran

**Keywords:** 3D printing, drop-on-powder, powder-based 3D printing, personalized medicine, 5-fluorouracil, Soluplus

## Abstract

This study reports the first case of an innovative drop-on-powder (DoP) three-dimensional (3D) printing technology to produce oral tablets (diameters of 10 mm and 13 mm) loaded with an anticancer model drug, 5-fluorouracil (FLU). For this study, a composition of the powder carrier containing CaSO_4_ hydrates, vinyl polymer, and carbohydrate was used as the matrix former, whereas 2-pyrrolidone with a viscosity like water was used as a binding liquid or inkjet ink. All tablets were printed using a commercial ZCorp 3D printer with modification. The resultant tablets were subject to coating with various polymeric solutions containing the drug. The composition of the polymeric solutions was adjusted at drug: polymer(s) 1:1 (*w*/*w*) ratio. Either Soluplus^®^ (SOL) alone or in combination with polyethylene glycol (PEG) was used to develop the coating solution of 2.5% (*w*/*v*) concentration. The particle size analysis, flow test, and particle morphology studies revealed mono-modal narrow size distribution, good flow properties, and porous loosely bound texture (of the tablets), respectively. Moreover, the advanced application of the fluorescence microscopy showed a homogenous distribution of the drug throughout the surface of the 3D printed tablets. The *in vitro* dissolution studies showed that the tablet compositions, dimensions, and the coating solution compositions influenced the release of the drug from the tablets. It can be concluded that our innovative DoP 3D printing technology can be used to fabricate personalized dosage forms containing optimized drug content with high accuracy and shape fidelity. This is particularly suitable for those drugs that are highly unstable in thermal processing and cannot withstand the heat treatment, such as in fused deposition modeling (FDM) 3D printing.

## 1. Introduction

Three-dimensional (3D) printing is an additive manufacturing technique, which is different from traditional machining techniques and has attracted a growing interest in rapid prototyping [1]. One of its major advantages is that it can fabricate complicated shapes and geometrics with higher possible shape fidelity and accuracy than the traditional fabrication techniques [2]. Nowadays, 3D printing is employed in medical devices, implants, tissue regeneration, pharmaceutical dosage form, and personalized medicine.

Powder-based (PB) 3D printing technology, also known as drop-on-powder (DoP) or binder jetting, was developed by the Massachusetts Institute of Technology (MIT) in the 1980s and was commercialized by Z-Corporation for producing different 3D printers [3]. It is regarded as the first technological adaption of 3D printing for pharmaceutical demands [2]. This process utilizes an ink-jet head that can jet-dispense a liquid binder solution onto a flattened powder bed. Then, particles of the powder carrier can be adhered together by organic or inorganic binders to form an agglomerated object because of adhesive forces or a hydraulic cement setting reaction [4]. In this event, the final 3D objects with desired geometry are created by stacking agglomerated layers in sequence. As a result, the produced oral dosage forms, such as tablets, are similar to fast-disintegrating tablets because they are comprised mainly of powder with loosely bound particles. Although there are many different 3D printing techniques suitable for pharmaceutical applications, the first Food and Drug Administration (FDA) approved 3D printed medicine (Spritam^®^) was developed through TheriForm^®^ technology, which is originally derived from a PB 3D printing technique [5]. Due to the printing nature, formulations, and process optimization, a 3D printed levetiracetam tablet has a highly porous internal environment and micron-scale pore size that can dramatically increase surface area, hence the ultra-rapid drug dissolution and release after oral administration [6]. 

Because of the benefits of its pharmaceutical application, such as easy adaptation and fixation, reduced development time, and favorable aesthetic results, DoP 3D printing technology can be better adapted to the manufacturing of dosage forms when compared to other 3D printing technology (i.e., fused deposition modeling (FDM)). One of the main reasons for this is that the starting materials (such as powders and binder solutions) have already been widely used in the pharmaceutical industry. However, there are some associated challenges, such as the additional drying process to eradicate residual solvents and to improve the physical resistance of the PB 3D printed constructs [2,7]. Still, it has immediate potential for unit dose fabrication. Moreover, with an appropriate process engineering and optimization, this possibly can be one of the first 3D printing processes suitable for fully scalable commercial exploitation. 

Therefore, we report a first case of a DoP 3D printing process to develop anti-cancer drug loaded tablets with varying diameters (10 mm and 13 mm). We used 5-fluorouracil (FLU) as a model drug to prove the concept of our printing process. This model drug is highly potent and available in low dosage. Though FLU is fairly heat stable, the successful optimization and subsequent development of its tablets using DoP heat-less 3D printing will provide for an ideal platform to process and evaluate many other model drugs that are highly thermo-sensitive, such as biologics or macromolecules. Optimized powder carrier containing CaSO_4_ hydrates were used as powder bed materials. The drug solution compositions were optimized using hydrophilic polymer combinations. This study proves that an optimized powder based 3D printing can also be used to develop pharmaceutical products using pharmaceutical grade excipients by eliminating the need for thermal processing. The latter can be a real issue for processing thermo-sensitive drugs. In addition, DoP is much easier and less time consuming than making filaments then using that filaments to fabricate tablets, as is the case in the FDM 3D printing.

## 2. Experimental Section

### 2.1. Materials

The model anticancer drug FLU with purity >98% was purchased from Hangzhou Longshine Bio-tech Co., Ltd. (Hangzhou, China). The printing CaSO_4_ powder was obtained from EMCO Education Ltd. (Portsmouth, UK). Soluplus^®^ was kindly donated by BASF, Germany. Polyethylene glycol (PEG) was procured from Sigma Aldrich (Gillingham, UK). All chemicals used were of analytical grade and used as received.

### 2.2. Experimental Methods

The tablets with diameters of 10 mm and 13 mm were printed using a ZCorp printer (Z-Corporation, Rock Hill, SC, USA) with a print layer thickness of ~100 µm. The tablet matrix was composed of a CaSO_4_ based powder, which was a mixture of CaSO4 (<90%) and vinyl polymer (<20%) with carbohydrate (<10%), whereas the liquid binding solution was an aqueous solution containing 2-pyrrolidinone, whose viscosity is similar to water. The approximate average weights of printed tablets with diameters of 13 mm were ~900 mg, and those with diameters of 10 mm were ~400 mg with an average thickness of 5 mm and 3 mm, respectively. The printing process only took <5 min for one tablet. The physical properties of the powder are listed in Table 1. The printing parameters such as binder volume, jet-dispensing speed, and drops fired were optimized and recorded throughout the printing process. The print head operated at a maximum temperature of 50 °C with a maximum current of 1.7 A and a fire voltage of 0.5 V, which was found to be optimal for the successful and smooth printing of the tablets. An AutoCAD design of the tablets was made using Solidworks (2017 SP5, Waltham, MA, USA) and transferred to the computer connected with the printer in stereolithographic (stl) file format prior to the actual printing process. The optimized composition of the pharmaceutical grade carrier powder utilized in the 3D printing process contained CaSO_4_ hydrates (<90% *w*/*w*) and vinyl polymer. After the printing process was finished, all tablets were subject to a quick drying in the build box before being removed from the powder bed. This drying process was conducted to ensure that the printed tablets were robust enough to withstand the handling process and that any redundant powders were removed easily after the cycle. Since the aim of this study was to prove the concept of DoP printing for the fabrication of anti-cancer drug loaded tablets, only the powder suitable for printing with Z-Corp (e.g., CaSO_4_) was used herein. This also eliminated any possibility for the intrusion of the performance and/or safety of the printer. Therefore, the incorporation of the drug in the tablet matrix was not performed at this stage. The coating aqueous solutions containing the drug FLU was optimized using hydrophilic polymers SOL and PEG. Various compositions were prepared as depicted in Table 2. The coating of the 3D printed tablets was performed using the drop-on-demand technique via utilizing micro-pipette and depositing the solution onto the surface of the tablet until a homogenous coating was achieved. As mentioned earlier, for the purpose of this proof of concept study, no additional materials except the suitable carrier powders were used in the printer. For this reason, the drug was added to the printed tablets by coating method.

Briefly, around 0.1 and 0.2 mL of the drug solution were dropped manually onto the surface of tablets (diameters of 10 mm and 13 mm) separately by using 20–200 μL Research^®^ plus pipette (Eppendorf Ltd, Stevenage, UK). After that, the tablet samples were put into the oven at 50 °C for 1 h. The drying process was finished until the weight of tablets would not decrease. 

The geometric primary particle size distribution of the printing powder was measured with a laser light diffraction analyzer (Helos/Rodos, Sympatec GmbH, Clausthal-Zellerfeld, Germany) equipped with the HELOS sensor and Windox software (version 5, Sympatec, Clausthal-Zellerfeld, Germany). Detection of the particles was carried out using the R3 and R5 lenses with the detection range of 0.5–175 μm and 0.5–875 μm, respectively. The surface morphology of the printed tablets was examined by Jeol JMS 820 (Freising, Munich, Germany) at the accelerating voltage of 3 kV. The samples were mounted on an aluminum stub using adhesive carbon tape and were sputter coated with gold under vacuum (Edwards S-150 sputter coater, Edwards High Vacuum Co. International, Albany, NY, USA). As fluorescence and UV-Vis irradiation are important photo-physical properties of FLU, the homogeneity of FLU on the surface of 3D-printed tablets was checked with an SP8 confocal microscope (Leica, Microsystems (UK) Ltd., Milton Keynes, UK). Also, the 3D-printed tablet without the drug solution was analyzed for a negative control. *In vitro* dissolution tests under sink conditions were performed with a US Pharmacopeia (USP) type II paddle apparatus (708-DS Dissolution Apparatus, Agilent Technologies, Santa Clara, CA, USA) in 900 mL of phosphate-buffered saline (PBS) (pH 6.8) at 37 ± 0.3 °C with a paddle speed of 100 rpm. All samples were run in triplicate (*n* = 3). The drug concentration of the dissolution medium was measured automatically by using Cary 60 UV-Vis (Agilent Technologies) at a wavelength of 265 nm in a 1 cm cell versus a blank solution consisting of a phosphate buffer (pH 6.8). Then, the release profiles were plotted as a percentage of cumulative drug release versus time. Additionally, there was no interference from Soluplus^®^ or PEG6000 on drug assay observed at the detection wavelength.

## 3. Results and Discussion

**Figure 1:**
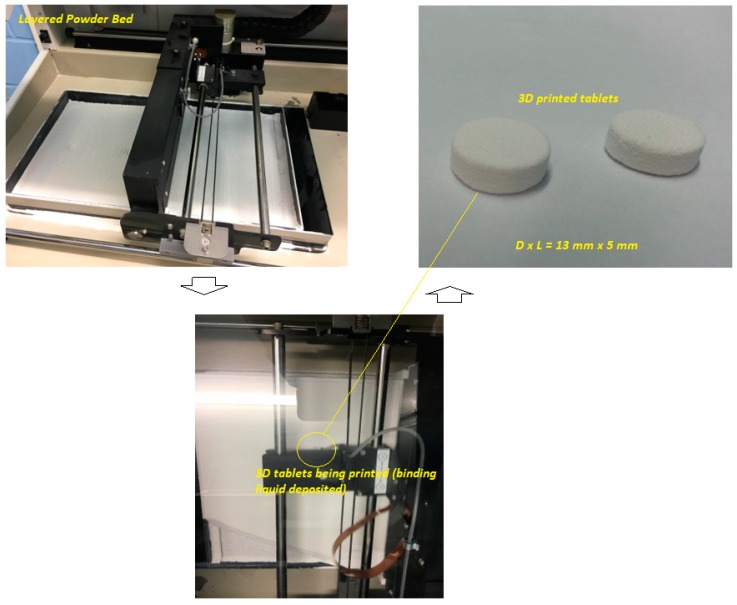
Schematic diagram of the printing process of drop-on-powder (DoP) three-dimensional (3D) printing process utilized in this study.

The powder-based 3D printing platform used the maximum layer resolution in accordance with the slicing fragments counted, which resulted in the estimated time for the printing of the whole series of the tablets at about 15–20 min (single tablet ~5 min). An interesting feature of this platform is that once the ideal formulation compositions and the processing parameters are optimized, this can be fairly scaled up for pilot scale manufacturing making it commercially viable process. The jet-dispensing rate plays a pivotal role in the texture and binding properties of the particles with the printed 3D objects (Figure 1). Our printing process was optimized so that all printed tablets were almost instantly ready for further evaluation, though an optional drying step can be introduced to produce tablets as robust as possible to withstand the handling process. This eliminates the wait-time for the eradication of the residual solvent. The process utilized an aqueous solution of 2-pyrrolidone, which is largely available in various pharmaceutical formulations [8]. The use of this binding liquid assisted in a smooth and faster printing process without any further post-processing steps. It has been reported that 2-pyrrolidone is an acceptable solvent to be used in drug delivery systems for humans. It is also widely present in foodstuffs and food additives [8]. 

**Figure 2:**
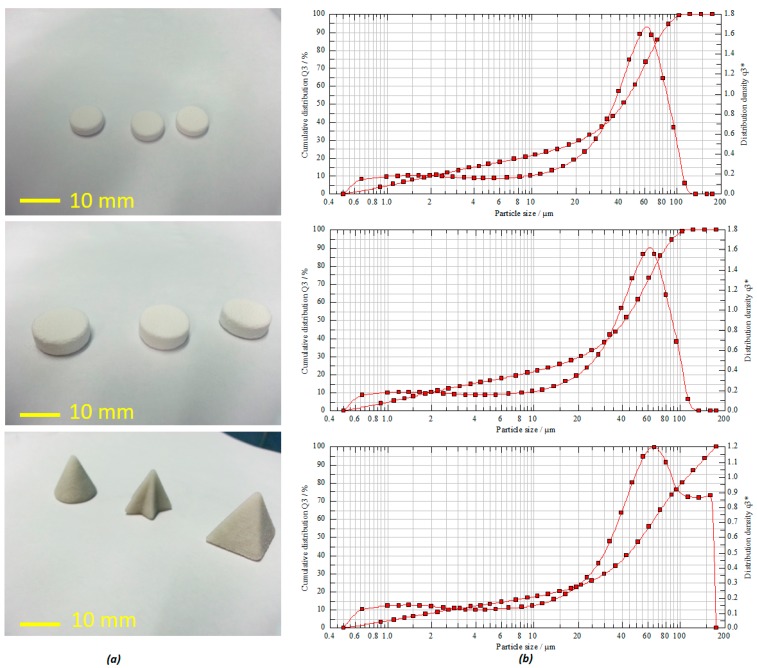
(**a**) 3D printed tablets with different shapes and dimensions; (**b**) volume-weighted particle size distribution (PSD) of the powder carriers used in the printing process.

The carrier powder was composed of pharmaceutical grade CaSO_4_ granules, vinyl polymer, and carbohydrate with narrow particle size distribution. The particle size and shape of active and non-active pharmaceutical ingredients in most pharmaceutical products can affect various significant physical properties and quality attributes, such as physicochemical stability and dissolution rate [9]. In Figure 2b, as the mode average diameter according to the peak is around ~50 μm, the flowability of these particles may just be at the threshold of good flow. This can sometimes result in incomplete layers appearing during the printing process [5]. However, in our process, despite the moderate to poor flowability and because of the process optimization and engineering, various tablets with high accuracy and shape fidelity were still printed successfully. 

The mean particle size distribution was found to be in the range of about 41.65–54.44 µm (*D*_50%_) with a volume mean diameter (VMD) of 41.46 µm (Figure 2b). The powder flowability test via both Carr’s index and angle of repose estimation for formulation powders exhibited good flow properties. This is reflected on the calculated values of the Carr’s index and angle of repose as 25 and 22.02 degrees, respectively. Moreover, the calculated bulk index value of the powder indicates that it rendered good compatibility as well (Table 1). It has been reported that the particle morphology of CaSO_4_ hydrates based powder is good for high-performance composite material for strong, accurate, and high-definition model making. In an optimized process, by simply spraying the suitable binding solution, this calcium sulfate hemihydrate (CaSO_4_ + *n*H_2_O) powder undergoes a self-hydration process, resulting in a semi-solid calcium plaster-like paste (CaSO_4_·$\frac{1}{2}$H_2_O + 1$\frac{1}{2}$H_2_O = CaSO_4_·2H_2_O) [4,10]. This process was adopted and repeated for each depositing layers in sequence until the 3D object was printed herein. As shown in Figure 2a, the tablets with various shape fidelity and dimensions were printed with high accuracy, uniformity, and reproducibility.

**Figure 3:**
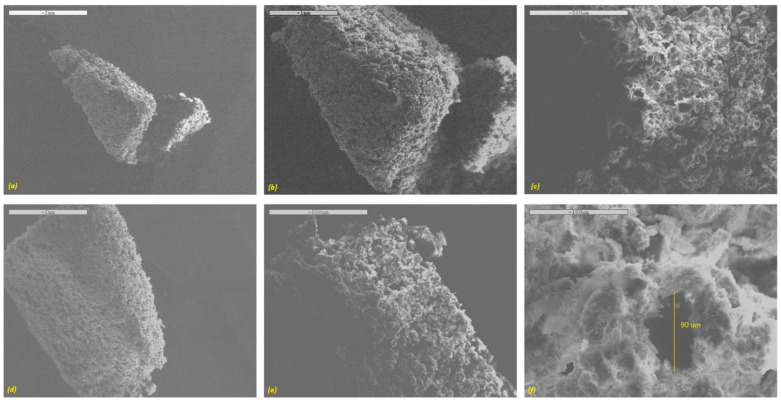
SEM images of the surface of DoP 3D printed tablets (**a**–**c**) 10 mm diameter and (**d**–**f**) 13 mm diameter.

The surface morphology of the printed tablets examined via SEM is depicted in Figure 3. It is obvious from the SEM images that all printed tablets had a highly porous surface with matrix forming particles loosely bound with the binding liquid. This can be ideal for the formulation of fast disintegrating dosage forms, such as orally disintegrating tablets (ODTs). All of the tablet formulations exhibited micron-scale interconnected pore size. A high-resolution SEM image revealed the pore diameter in the region of about ~90 µm (Figure 3f). As a result, upon contact with an aqueous medium such as a dissolution solution, the surface area could be increased significantly, resulting in faster dissolution of the drug from the tablets.

The model anti-cancer drug, FLU, is sparingly soluble in water and slightly soluble in ethanol [11]. However, the solubility of FLU can be affected significantly by temperature, and it can be increased more than 12-fold by enhancing the temperature between 25 and 200 °C under a constant pressure of 5.1 MPa [12]. Additionally, Singh et al. pointed out that FLU has high thermal stability when the temperature is less than 278 °C [13]. Having considered this property, during the preparation of the drug-loaded coating solution, the temperature was increased to around 70 °C (i.e., in case of solution C (2.5% *w*/*v* FLU)) in order to increase the concentration of the drug in solution. As a result, the maximum concentration of the solution was optimized at 25 mg/mL.

Soluplus^®^ (SOL) is a graft co-polymer of polyvinyl caprolactam (PVC)-polyvinyl acetate (PVA)-polyethylene glycol (PEG) that can help drug dispersed molecularly in its matrix in the preparation of solid dispersion. As a result, Thakral et al. proved Soluplus^®^ can increase the water solubility of an anti-cancer drug with poor aqueous solubility—camptothecin—in a colon-targeted delivery system [14]. Their solid dispersion formulation of camptothecin in Soluplus^®^ with citric acid had great potential for colorectal cancer therapy. Uddin et al. studied Soluplus^®^ as a drug carrier and the coating formulations (consisting of drug-polymer solutions at various ratios) [15]. They found that Soluplus^®^ helped the drug release for various anti-cancer substances (5-fluorouracil, curcumin, and cisplatin), especially for the water-insoluble drugs (curcumin and cisplatin) because of their solubilizing enhancement capacity. In addition, Homayouni et al. pointed out that this polymer can perform as a stabilizer and a solubilizing agent in poorly water-soluble drug formulation [16]. Because Soluplus^®^ contains a polyethylene glycol backbone as the hydrophilic portion and vinyl caprolactam/vinyl acetate side chain as the lipophilic part, the significant amphipathic property gives it good surface activity, wettability, ability to enhance the aqueous solubility, and oral bioavailability [17,18]. As a result, all developed polymeric solutions (SOL alone or in combination with PEG) contained FLU dispersed homogeneously within the matrices. 

**Figure 4:**
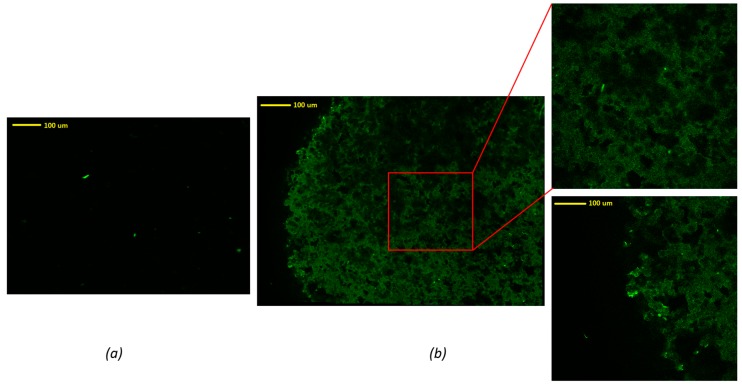
Confocal microscopic images of (**a**) tablets without drug solution, (**b**) tablets with drug solution C.

In order to evaluate the homogeneity of the drug distribution upon depositing onto the formulated tablets, an advanced reverse optical microscopic analysis was conducted. As can be seen in Figure 4, an uncoated tablet without drug solution exhibited no fluorescence traces in the mapping image (Figure 4a). There were some bright green dots that may be attributed to some degree of contamination during the sample preparation or handling. These green dots were also present in the FLU loaded tablets, indicating that these are not relevant to the presence of the drug. It could also have been due to the presence of dust on the surface of the tablet. However, the images of tablets coated with drug solution showed significant differences compared to the tablet without drug solution. In contrast, the FLU loaded tablet surface showed a homogenous distribution of the drug throughout the surface of the tablets, represented by the dark green pattern. From these images, the edge of the tablet can be figured out easily. It can be concluded that the drug was coated homogeneously on the surface of these 3D printed tablets.

**Figure 5:**
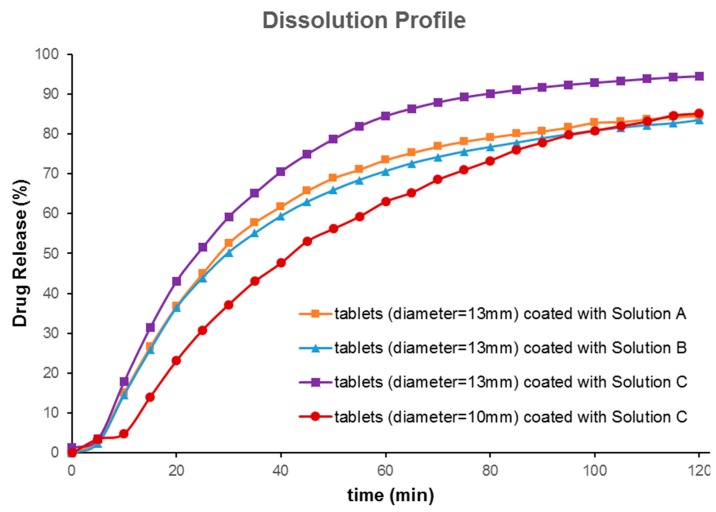
*In vitro* drug release profiles (under sink condition) of various printed tablets coated with solutions A, B, and C containing 5-fluorouracil (FLU) at pH 6.8 (*n* = 3).

*In vitro* drug release profiles from both 10 mm and 13 mm tablets coated with FLU solution A, B, and C for 2 h are depicted in Figure 5. Tablets with a bigger diameter (13 mm) showed better dissolution behavior when compared with small tablets (Figure 5a). The probable reasons may have been the larger volume of 3D printed tablets for absorbing the drug solution and the larger surface area for partial hydration reaction in the dissolution media. The dissolution test was carried out for 6 h; as over 80% of the drug was released within the first 120 min, only data up to 120 min were picked up for further evaluations. The aim of the current research was not to show sustained release behavior of printed tablets. Rather, it was to show that the drug release from printed tablets can be modified to reach the dissolution profile needed. Asadi-Eydivand et al. pointed out that calcium sulphate hydrated powder could absorb moisture from the environment [10]. The comparison graphs of solutions A, B, and C clearly illustrate that all these drug solution coated tablets showed a sustained release over 2 h. Tablets coated with solutions A and B showed slightly slower release compared to that of solution C, which contained only FLU. About 90% of the drug had been released from polymeric solutions, whereas the bulk FLU solution showed about 100% release in 2 h. The slight delay in the release of the drug from the polymeric solution could be attributed to the chemistry of amphiphilic polymer, Soluplus^®^. It has been reported that Soluplus^®^ tends to retard the release of the sparingly water-soluble drug upon swelling in the dissolution media [19]. Comparing the dissolution profiles of the 13 mm and the 10 mm tablets coated by solution C showed that these dissolution profiles were different (*f*_2_ value of 43; *f*_2_ value (similarity test); less than 50 indicates that they are different). This indicates that the size of printed tablets has a significant effect on the dissolution profiles of FLU. Comparing the effect of the coating solution showed that the type of solution cannot make a big difference (similarity factor *f*_2_ was higher than 50). Nonetheless, our developed formulation compositions (solutions A and B) could be ideal for prolonging the release of the drug for a longer time or sustained release drug delivery, as may be required for chemotherapeutic drug delivery systems. 

## 4. Conclusions

The authors of the current research successfully demonstrated that an optimized DoP 3D printing process can be applied for the fabrication of oral dosage forms, such as tablets with varying shapes and morphology. A detailed investigation of the powder compositions revealed that the particle morphology, flow, and compression properties play pivotal roles in powder based printing processes. The produced tablets were also coated with anticancer model drug solutions, where drug particles were seen homogenously dispersed throughout the surface of the printed tablets. Furthermore, it could be claimed that the DoP 3D printing process demonstrated here was able to adjust the dose, release rate, and loading of the drug substances by changing the formulation composition and the processing parameters. This method can be further scaled up for pilot scale manufacturing in the personalized medicines arena. In conclusion, it can be claimed that DoP 3D printing process reported herein can be exploited for a number of drugs, including thermolabile drugs, by optimizing the powder bed composition using pharmaceutical grade excipients such as polymers. This will indeed present this emerging technique at the forefront of additive manufacturing to the delivery of immediate potential for unit dose fabrication of personalized medicines. 

## Figures and Tables

**Table 1:** Properties of the powders used for printing matrices.

Printing Powder Carrier Properties				
Viscosity(cP_0_)	Flowability		Bulk Density (g/cm^3^)	*D*_50%_, *D*_90%_ (µm)
	Carr’s Index	Angle of Repose		
220.8 (Torque: 18.4% Speed: 5.00 rpm)	25	22.05°	1.172	42.32, 80.12

**Table 2:** Drug coating solution compositions.

Tablets	Excipients	Solution A	Solution B	Solution C
Tablet (batch 1) (Diameter = 10 mm)	5-fluorouracil (mg)	2.5	2.5	2.5
	Soluplus (mg)	2.5	1.25	0
	Polyethylene glycol (PEG) (mg)	0	1.25	0
Tablet (Batch 2) (Diameter = 13 mm)	5-fluorouracil (mg)	5	5	5
	Soluplus (mg)	5	2.5	0
	PEG (mg)	0	2.5	0
